# EU Snapshots: 2021 Matters Pandemic and Endemic

**DOI:** 10.1111/jcms.13409

**Published:** 2022-08-14

**Authors:** Nikolaos Gkotsis‐Papaioannou

**Affiliations:** ^1^ University of Surrey, Guildford

Presidencies of the EU Council 2021: Portugal (1 January–30 June) and Slovenia (1 July–31 December)

Subsequent presidencies 2022: France (1 January–30 June) and Czechia (1 July–31 December)

**January**

4: British judge rejects US request to extradite WikiLeaks founder Julian Assange, stating mental health concerns.
4: A Norwegian firm (DNV GL) that was meant to have provided technical and safety certificates for Russia's Nord Stream 2 pipeline system to Germany has abandoned the project due to US sanctions.
4: The European Commission states that it is examining the preliminary agreement reached by the UK and Spain regarding Gibraltar. The deal would allow the British Overseas Territory to join the Schengen area, with Spain ‘ultimately responsible’ for controls at Gibraltar's port and airport.
5: As coronavirus cases continue to increase, UK PM Boris Johnson declares lockdown for England until mid‐February. On the same day, the German government and the majority of Germany's federal states agree to extend lockdown measures until 31 January.
5: Britain records a staggering 1,041 people daily death toll, the highest since 8 April. The numbers made Britain Europe's worst corona‐hotspot, ahead of Italy.
6: European leaders express distress as armed supporters of US president Donald Trump storm the Capitol building in Washington in an effort to stop lawmakers from confirming president‐elect Joe Biden's electoral win. Trump supporters, who were earlier urged by Trump at a rally to go to the Capitol, stormed the building as Congress members were certifying Biden's win.
6: After consultation with the 27 EU member states, the European Medicines Agency (EMA) authorizes the use of the Covid‐19 vaccine developed by US company Moderna.
6: The Netherlands begins its Covid‐19 vaccination programme, almost two weeks after most other EU countries. Belarus starts a coronavirus vaccination drive this week using the Sputnik V jab, becoming the first country outside Russia to use the vaccine developed by Moscow.
9: Italy's far‐right former interior minister, Matteo Salvini, appears before a judge in Palermo, Sicily, regarding his order to block migrants from disembarking in August 2019.
11: Britain authorizes the use of thiamethoxam, a bee‐killing pesticide banned by the EU in 2018, pursuant to lobbying by the National Farmers' Union and British Sugar advocacy groups. UK authorities respond that some states have approved ‘emergency’ use of the product despite the EU decision.
11: The EU anti‐fraud office Olaf launches an investigation into the EU's border agency, Frontex.
13: Estonia's ruling coalition, including the far‐right and anti‐EU EKRE party, collapse after the resignation of Centre‐party PM Juri Ratas when senior staff were named as suspects in a corruption inquiry.
14: The ECJ issues historic opinion in a case heard in the Irish language, regarding a complaint by Peadar Mac Fhlannchadha, who wanted dual‐language labels for his dog's medicine, in accordance with his EU rights.
15: The four coalition parties of the Dutch government under PM Mark Rutte resign two months before planned elections.
16: North Rhine‐Westphalia state premier Armin Laschet is elected as the new leader of Germany's ruling party, the Christian‐Democrat Union (CDU), and the successor of current German chancellor Angela Merkel.
19: According to UN agencies, approximately 43 people drowned trying to leave Libya for the EU by boat in the first lethal incident of its type in 2021.
24: Portuguese far‐right party Chega comes third in Portuguese presidential elections with 11.9 per cent of the vote, up from 1.3 per cent in a parliamentary election in 2019. The centre‐right president Marcelo Rebelo de Sousa is re‐elected with 60.7 per cent, ahead of Socialist Ana Gomes on 12.9 per cent.
25: Germany's far‐right AfD party files two lawsuits designed to stop German intelligence service, the BfD, from designating it as a threat to national security and putting it under surveillance.
26: The International Monetary Fund (IMF) predicts the global economy is set to lose over €18 trillion between 2020 and 2025, due to the impacts of the coronavirus pandemic.
27: Kaja Kallas is sworn in as the first female PM since Estonia's independence in 1991, following a coalition deal between her centre‐right Reform party and the left‐wing Centre party.
27: The UK offers EU nationals living in the UK up to £2,000 plus the cost of flights home if they would rather undergo a ‘voluntary return’ than apply for ‘settled status’.
27: Poland brings a divisive anti‐abortion law into force, amid escalating EU doubt on the legality of its court system. The law bans all terminations except for cases of rape and incest or if the mother's life was at risk.
29: Hungary sees the sharpest drop among EU countries in the annual corruption index compiled by Transparency International (TI), with a drop of 11 points since 2012, scoring 44 out of 100 on the index.
29: Serbian president Aleksandar Vučić announces a vaccination programme in Kosovo without consulting leaders in Pristina or specifying which vaccine.
29: Hungary's drug regulator approves China's Sinopharm Covid‐19 vaccine, making the country the first in the EU to do so.
29: The Commission adopts an export control mechanism on vaccines produced in EU countries, as a response to the sudden announcement of shortfalls by the pharmaceutical company AstraZeneca.
31: Belgian police arrest more than 200 people at an anti‐lockdown protest near Brussels' Midi train station. Sizeable demonstrations also took place in Budapest and in Vienna, where they were organized by Austria's far‐right FPÖ party and included neo‐Nazi militants.
31: Several EU leaders and EU Commission president Ursula von der Leyen condemn the military coup in Myanmar.

**February**

1: The European Centre for Disease Prevention and Control (ECDC) launches its Covid‐19 vaccine tracker, a dashboard that provides the latest data about vaccination programmes in member states.
1: A delegation of more than 70 representatives from Libyan regional powers heads over to Geneva for five days to select an interim PM in an EU‐ and US‐backed UN process.
1: According to *The Times*, British diplomats are to carry out security vetting of Chinese academics who intended to do research in the UK in ‘sensitive’ subjects.
2: Following a heated dispute over delayed deliveries, vaccine developer AstraZeneca and Pfizer‐BioNTech are expected to supply extra doses to EU member states in the second quarter.
2: Mevlüt Çavuşoğlu's remarks after talks with Turkish‐Cypriot leader Ersin Tatar that reunifying ethnically divided Cyprus based on a federal formula is off the table in any future peace talks and any deal should be negotiated between two equal sovereign states.
3: The ECJ rules that Hungary ‘manifestly failed’ to adopt timely measures to tackle pollution as required by the timeframe set out in the EU law on clean air.
3: The Commission tasks the EU agency for cybersecurity, ENISA, with creating an EU‐wide certification scheme for 5G networks.
4: Belgium joins Denmark, Germany, Greece, Hungary, the Netherlands, Romania, Slovenia and Sweden as an EU ‘hub’ state for storing extra medical equipment in the pandemic.
5: Russia expels three European diplomats as they attended ‘unlawful’ rallies in support of jailed opposition hero Alexei Navalny. The decision was announced during a press conference between EU top diplomat Josep Borrell and Russian foreign minister Sergei Lavrov.
6: Residents on the Greek island of Chios continue to protest plans for the creation of a new closed migrant camp.
7: European charity SOS Méditerranée boat Ocean Viking rescues 422 migrants off the coast of Libya and states that it received permission from Italy to disembark in the Sicilian port of Syracuse.
8: Germany, Sweden and Poland each expel a Russian diplomat following Russia's dismissal of three diplomats from those EU member states the previous week.
8: Eurostat indicates nearly 29 per cent of the EU population with a disability (aged 16 or over) was at risk of poverty or social exclusion in 2019, compared to 18.4 per cent of those with no activity limitation.
10: The trial of French far‐right leader Marine Le Pen starts in Paris. Charges brought against her relate to breaching hate speech laws by tweeting pictures of atrocities committed by the Islamic State in 2015. Le Pen's defence argues the charges challenge her right to free speech.
10: A Dutch court suspends extradition of a suspected drug trafficker to Poland (under the European Arrest Warrant) citing concerns for political control over Polish judges.
11: Foreign ministers and senior officials from Saudi Arabia, Bahrain and the United Arab Emirates meet with foreign ministers from Greece, Cyprus and Egypt in Athens. This is part of Greece's efforts to expand alliances to counter tension with Turkey.
14: A bloc of pro‐independence parties win 51 per cent in Catalonia's devolved parliament elections. The centre‐left Catalan Socialist party came first overall (23 per cent), the far‐right Vox party 11 per cent, while centre‐right parties struggled. This victory triggers renewed calls for dialogue between Madrid and Barcelona for a political solution to the independence conflict.
14: Anti‐Serbian, left‐wing Albin Kurti and his allies win parliamentary elections in Kosovo, gaining almost 48 per cent of the votes.
15: The Commission urges Hungary to let one of the last independent radio broadcasters, the government critical Klubradio station, to keep going after it lost its licence. Klubradio was being forced off air on ‘highly questionable legal grounds’ in what would cause it ‘irreparable damage’ regarding ‘media freedom and pluralism’.
17: Digital and human rights organizations, led by European Digital Rights, launch a European Citizens' Initiative calling for a ban on the use of biometric mass surveillance in public spaces.
17: Italy's new PM Mario Draghi calls on Italians for unity to help rebuild the country following the coronavirus pandemic and promises his new government would introduce sweeping reforms to revitalize the economy.
18: The Commission launches legal action over Hungary's failure to implement a judgment from the ECJ, which ruled that legislation on restricting foreign funding of NGOs was against EU rules.
22: Almost two dozen MEPs raise concerns over Polish anti‐LGBTi influence within the European Economic and Social Committee (EESC).
22: The EU agrees to blacklist Russian officials guilty of wrongly jailing opposition figure Alexei Navalny, making first use of the Magnitsky Act on Russia, as well as to enforce asset freezes and visa bans.
23: The UK government agrees to allow the EU more time to formally ratify the post‐Brexit trade deal. The provisional application is extended until 30 April.
23: Greek doctors proceed with a day‐long strike, with some in Athens also marching in protest at what OEGNE, the medical trade union, called ‘suffocating’ conditions on intensive‐care wards, which were 80 per cent full due to the pandemic. Doctors call for segregated wards for corona patients and extra funds.
24: Turkey accuses Greek jets of ‘harassing’ a scientific research vessel in the Aegean Sea in claims denied by Athens. The two countries are currently preparing to resume de‐escalation talks.
25: Authorities in Germany and Belgium seize 23 tonnes of cocaine bound for the Netherlands, in what is regarded Europe's biggest haul.
25: The head of Frontex, Fabrice Leggeri, welcomes European Parliament scrutiny of the under‐fire agency and suggests such a probe should have already been launched.
25: The ECJ fines Spain €15 million plus a daily penalty of €89,000 for failing to fulfil its obligations under the directive on protection of personal data during police investigations.
26: Malta concludes its investigation into the 2017 murder of journalist Daphne Caruana Galizia, after one of three alleged hitmen – Vince Muscat – pleaded guilty.

**March**

1: Germany tightens border controls on France's eastern Moselle region as a ‘variant of concern’ area, triggering tougher entry requirements at the border between the two neighbours.
1: Former French president Nicolas Sarkozy is given a prison sentence after being found guilty of corruption and influence peddling.
1: Czech PM Andrej Babiš tightens lockdown measures, as the country reaches the highest‐per‐capita infection rate in the world over the past week.
1: EU foreign relations chief Josep Borrell tells MEPs that his service does not have the resources to counter Chinese and Russian disinformation on the same, huge scale that it is being used against Europe.
3: The Commission presents a strategy to strengthen the rights of people with disabilities for the period 2021–30. This will include an EU disability card to ensure the mutual recognition of disability status between member states by the end of 2023 and an initiative to improve social services for people with disabilities.
4: MEPs hold a public cross‐examination with the head of the EU's border agency Frontex, Fabrice Leggeri, as part of a wider European Parliament probe known as the Frontex Scrutiny Working Group. The initial draft agenda proposed to hold it behind closed doors.
4: German intelligence is to carry out surveillance on the far‐right AfD party (excluding AfD candidates taking part in the regional and federal elections later this year), amid fears its extremist wing was gaining influence.
4: Germany, Poland and Switzerland offer help with treating Covid‐19 patients in the Czech Republic, after a surge in infections.
5: The Commission publishes a study, which finds that the pandemic has proven to be a major challenge for gender‐equality across Europe, with domestic violence and existing inequalities exacerbated.
7: A far‐right proposal to ban facial coverings in Switzerland passes by a narrow margin in a binding referendum. The proposal does not mention Islam directly, but local politicians, media and campaigners have dubbed it the ‘burqa ban’.
9: The European Parliament votes to lift parliamentary immunity of Catalan MEPs.
10: Financial products firms, such as insurers and pension funds, who market themselves on backing environmentally friendly investments, will face tougher reporting requirements as the EU's Sustainable Finance Disclosure Regulation (SFDR) enters into force. The SFDR regime aims to divert €1 trillion into more sustainable sectors and to prevent ‘greenwashing’.
10: European Parliament president David Sassoli, Commission chief Usula von der Leyen and Portugal's PM António Costa sign a joint declaration on the Conference on the Future of Europe.
14: Despite the EU's previous warning, Kosovo opens an embassy in Jerusalem making it the first majority‐Muslim country to recognize the contested city as Israel's capital. The EU previously cautioned against this action as it contradicted EU foreign policy.
15: Europe faces a third wave of coronavirus infections, amid spikes in numbers across the EU.
15: The Commission launches legal action against the UK over what Brussels says are breaches of the Brexit withdrawal agreement's arrangements in Northern Ireland.
15: The chief of the surveillance section at ECDC, Bruno Ciancio, warns during a public hearing in the European Parliament's health committee that most countries in Europe are falling short on tracking coronavirus variants. This means that more contagious strains could be spreading undetected.
15: Ireland enters the Schengen Information System (SIS).
16: Germany, France and Italy join the Netherlands and Ireland in suspending the Oxford/AstraZeneca's Covid vaccine, hours after the WHO announced it had seen no evidence the shot had caused blood clots in some people who received it.
16: Hungary and Poland win separate tax disputes with the Commission after the CJEU ruled in their favour.
16: Britain, the EU and Norway reach a three‐way deal regarding fishing quotas within the North Sea in the wake of Brexit.
17: Dutch centre‐right PM Mark Rutte is poised to extend his 10‐year rule after elections which also saw gains for new liberals and the far‐right.
19: Two weeks after Hungary's ruling Fidesz party's MEPs left the European People's Party (EPP), the party also leaves the larger political family.
19: The G7 group ‘unequivocally denounce Russia's temporary occupation of the autonomous Republic of Crimea and the City of Sevastopol’.
19: The EU freezes plans to sanction more Turkish officials over gas drilling in Cypriot‐claimed waters, amidst a detente in relations.
22: EU foreign ministers blacklist four Chinese officials over ‘serious human rights violations’ against the Uyghur minority, in their first sanctions on Beijing since the Tiananmen Square massacre of 1989.
22: Britain blocks holidays abroad until at least July to avoid Europe's third wave of coronavirus.
23: Over 250 MEPs and national parliamentarians urge the EU to support a vaccine patents temporary waiver of certain obligations under the Agreement on Trade‐Related Aspect of Intellectual Property Rights (TRIPS).
25: German interior minister Horst Seehofer announces easing of citizenship rules for Nazi victims' relatives.
25: The European Parliament endorses with a plenary vote the initiation of EU accession talks for North Macedonia and Albania. Similar support was expressed from the Commission.
26: EU leaders offer more refugee aid and customs perks to Turkey at a video‐summit also attended by US president Joe Biden.
29: EU countries extend the mandate of ‘Irini’, a naval mission to stop arms smuggling to Libya and human trafficking to Europe, until 2023.
29: The huge container ship, MV Ever Given, is being refloated from the banks of the Suez Canal, almost a week after the blockage.

**April**

1: The EMA insists that the AstraZeneca vaccine's benefits outweigh the risks but acknowledges that people should be aware of the ‘remote possibility’ of rare blood clots occurring.
1: President Emmanuel Macron orders France into its third national lockdown.
2: WHO regional director for Europe, Hans Kluge, criticizes Europe's vaccination campaign as ‘unacceptably slow’ while rising infection rates in most countries across the region mean its virus situation is increasingly alarming.
2: Dutch caretaker‐PM Mark Rutte narrowly survives a vote of confidence over his conduct in coalition‐forming talks by MPs.
4: Parliamentary election results find Bulgaria's incumbent PM Boyko Borissov winning but with a weaker public mandate due to corruption allegations.
6: Greece accuses a Turkish coastguard vessel of twice crashing into a Greek port‐authority boat in a recent incident and other Turkish ships of pushing migrant dinghies towards Greece. The reports come amid EU efforts to reset relations with Turkey, including new EU funds for refugees.
6: Deutsche Welle reports online leak of data from hundreds of millions of Facebook users, including personal information.
7: Ukraine's president Volodymyr Zelensky urges NATO to speed up his country's path towards membership, arguing there is no other way to prevent further conflict in the eastern Donbas region.
8: The European Court of Human Rights (ECtHR) finds that compulsory vaccinations are legal in a ruling that might create a precedent for Covid‐19 vaccination programmes.
9: French president Emmanuel Macron pledges to close the École nationale d'administration (ENA), an elite French academy leading to top civil service posts, in response to popular anger against social inequality, ahead of next year's elections.
12: The UK lifts its coronavirus lockdown, reopening non‐essential shops, gyms and recreational spaces with health precautions in place.
13: The EU refuses to help Montenegro pay off its almost €1 billion loan from China.
13: The European Investment Bank (EIB) is to manage €5 billion of the €32 billion that Greece will receive in grants and loans from the EU's pandemic recovery fund. This amount will be directed towards green and digital‐sector investment.
19: Russia declares 20 Czech diplomats ‘personae non grata’ following a similar move by Prague. Czech authorities expelled 18 Russian diplomats two days prior, accusing them of being secret agents.
19: The co‐leader of Germany's Greens, Annalena Baerbock, announces her run for chancellor in September's elections. This is the first time the party has sought this position in its 40‐year history.
19: EU foreign affairs chief Josep Borrell says no new Russia sanctions were being prepared, despite its largest‐ever deployment of 150,000 troops on Ukraine's borders, and the risk of igniting a wider conflict.
22: Hungary's government announces plans to amend laws that underpinned its attacks on foreign‐funded universities and civil society organizations, which the ECJ found as breaking EU rules.
23: The EU launches legal action against the pharmaceutical multinational AstraZeneca for failing to meet its contractual obligations for the supply of Covid‐19 vaccines, and for lacking a ‘reliable strategy’ to ensure timely deliveries.
26: Italy joins other countries by imposing restrictions on travel from India to avert the spread of a Covid‐19 variant.
28: The European Parliament votes overwhelmingly in favour of the post‐Brexit trade agreement between the UK and the EU, clearing a key hurdle in the ratification process.
29: Reuters reports the UK's plan to use a National Health Service phone application as its Covid‐19 vaccine certificate to facilitate travel internationally during the summer.
29: Arlene Foster announces she is stepping down as leader of the Democratic Unionist party and Northern Ireland's first minister after a revolt by party hardliners.
30: Jonas Grimheden is announced to become Frontex's fundamental rights officer, amid intense scrutiny over the agency's long delay in hiring staff tasked to ensure rights violations are avoided.

**May**

3: The Polish government extends the operation of a controversial open‐pit coal mine (Turów) until 2044, despite it breaching EU laws according to the Commission.
4: The last of the two model refugee camps on the Greek island of Lesbos closes, with around 1,000 people hosted there being sent to dire conditions in Moria.
4: Authorities in Germany take down one of the world's biggest child sex‐abuse platforms (‘Boystown’), following a large‐scale investigation which led to several arrests in mid‐April.
4: According to official statistics, the number of crimes committed by far‐right extremists in Germany jumped to the highest level ever recorded in 2020.
6: Madrid's People's party (PP) leader Isabel Diaz Ayuso, a fierce critic of Covid‐19 lockdowns, secures a major victory in regional elections. Spanish far‐right party Vox will help form a regional government.
6: French fishers threaten to blockade ports in the Channel Islands, in an escalation of a post‐Brexit row in which the French maritime minister has backed calls to cut off Jersey's electricity supply.
8: More than 1,000 migrants arrive on the small Italian island of Lampedusa prompting new political tension. The Commission subsequently demanded EU states to come to Italy's aid.
10: According to an investigation by *The Times*, Prince Michael of Kent and his associate, the Marquess of Reading, have been peddling their alleged influence in Russia in return for fees of €11,000 a day to foreign companies.
11: *The Guardian* reports that due to a backlog of more than 320,000 applications for post‐Brexit residency status, hundreds of thousands of European citizens could find themselves in limbo after 30 June, without documented legal rights to remain in the UK.
11: Josep Borrell announces the EU's intension to issue a statement of support for pro‐democracy activists in Hong Kong even without unanimous agreement of member states – Hungary vetoed a draft communiqué.
12: Stefan Yanev is appointed as leader of a caretaker Bulgarian government until new elections, in an effort to quell a political crisis. Although former PM center‐right Boyko Borissov's GERB party secured most seats in the April election, he failed to produce a viable coalition government.
17: Following the Israeli bombing raid that laid waste to the offices of the Associated Press (AP), Al‐Jazeera and other international media, the EU criticises Israel's rocket attacks as ‘extremely worrying’.
17: The Commission appoints former basketball player Michaela Moua as its first ever anti‐racism co‐ordinator.
17: More than 5,000 migrants enter from Morocco into the Spanish exclave of Ceuta. Spain has decided to return some 2,700 migrants. Spain has a deal with Morocco to expel migrants en masse without hearing asylum claims, except for unaccompanied minors who are allowed to stay.
18: Hungary, once again, undermines unanimity in the EU's call urging a ceasefire in Gaza.
18: The European Parliament votes to spend €17.5 billion on making the EU economy more ‘green’ by a majority of 615 against 35.
19: At least 57 migrants drown and 33 are rescued in a shipwreck off Tunis as they tried to cross the Mediterranean from Libya to Italy.
20: According to a survey by EU agency Eurofound, trust in national governments fell sharply in Austria, Bulgaria, Croatia, the Czech Republic, Cyprus, Greece and Poland during the pandemic. People in France, Hungary, Malta, Portugal, Romania and Spain also trusted EU authorities more than their own administrations.
21: Hungary vetoes a new trade and development deal with 79 African, Caribbean and Pacific countries saying it would open the door to migrants.
23: Belarus president Aleksander Lukashenko forces a passenger flight from Athens to Vilnius, carrying more than 140 people onboard, to make an emergency landing in Belarus instead. Commission president Ursula von der Leyen demands ‘Ryanair hijacking must be sanctioned’ and the opposition blogger on board who was snatched must be freed. EU leaders issue a joint statement agreeing to a first wave of economic sanctions against Belarus and threatened there were more to follow.
26: Switzerland decides to terminate the negotiations on the EU–Swiss framework agreement.
26: Ireland becomes the first EU country to formally designate Israeli settlement expansion as ‘annexation’ of Palestinian land.
29: More than 1,000 people protest mask‐wearing and vaccines in Brussels.

**June**

1: After two decades of legal and political battles, the EU public prosecutor's office (EPPO), responsible for uncovering and prosecuting fraud against the EU budget, starts its operations. The Luxembourg‐based independent office is headed by former Romanian anti‐corruption chief, Laura Kövesi.
2: Opposition mayors in Budapest rename streets after the Dalai Lama, ‘Free Hong Kong’, ‘Uyghur Martyrs’ and a Chinese bishop around the site where PM Viktor Orbán's government plans to build a campus for China's Fudan University.
2: The Commission announces the EU's intention to keep the deficit rules suspended throughout next year as well as for 2021.
3: EU ambassadors add Japan to a list of safe countries ahead of the Olympic Games.
3: A Commission study reveals an alarming increase in antisemitic online content during the pandemic.
3: The socialist‐led coalition government in Denmark passes a new law, effectively outsourcing asylum to countries outside Europe. The Commission condemns this bill as not compatible with EU law.
4: An ECJ ruling finds Germany persistently violated EU limits on air pollution.
4: The EU submits to the World Trade Organization (WTO) a plan aimed at expanding the production of Covid‐19 vaccines. Brussels sees this as a quicker and more‐targeted solution to unequal global distribution of vaccines than the intellectual property right‐waiver proposal backed by the US.
7: Finance ministers in London from the G7 group of wealthy nations agree a landmark deal aimed at making the biggest companies such as Apple, Microsoft, Google and Facebook pay more tax.
7: EU member states give final approval to the €17.5 billion Transition Fund, intended to support the transition of currently fossil fuel‐dependent regions to green their economies.
7: Slovakia becomes the second country in the EU to vaccinate its citizens with the Russian‐made vaccine Sputnik V.
9: The Commission launches legal action against Germany, after determining that last year's controversial ruling on bond‐buying by the country's Constitutional Court ‘constitutes a serious precedent’ that puts the EU's legal order at risk.
9: The European Parliament approves the EU's Covid travel pass.
14: Thousands of people join protests in Madrid against controversial plans to pardon jailed Catalan separatists.
15: The Hungarian parliament passes legislation which bans what it deems the ‘promotion’ of homosexuality among children, amid fierce criticism from human rights groups and most opposition parties.
16: The EU's Fundamental Rights Agency (FRA) warns in a published report that even though more than 300 million doses of Covid‐19 vaccines have been administrated in the EU, vulnerable groups, such as prisoners, homeless people and migrants, are at risk of missing out.
17: Northern Ireland's biggest parties agree to support a replacement for former first minister Arlene Foster. Her successor, Edwin Poots, is seeking to nominate his social conservative colleague Paul Givan to take over as first minister.
17: The Commission approves Greece's plans on spending €30.5 billion from the EU's post‐pandemic recovery plan.
18: The European Ombudsman delivers a verdict after a six‐month probe into Frontex. The probe found that only 22 complaints had been filed since 2016, suggesting ‘lack of awareness, fear of negative repercussions or lack of engagement by deployed Frontex officers’.
21: Transparency International reports three out of 10 people in the EU pay a bribe or use a personal connection to access a public service. The Global Corruption Barometer report for the EU finds two‐thirds of EU citizens surveyed consider corruption to be a considerable problem for their public institutions. More than half think their governments are controlled by private interests and that institutions have not been transparent during the ongoing health crisis.
21: The Swedish parliament passes a vote of no confidence in PM Stefan Löfven – it is the first time in Sweden's history that a PM has been ousted in such a vote.
24: Research by the advocacy group Avaaz reveals that Facebook is considered the biggest ‘emitter’ of fake news related to the pandemic. YouTube and Twitter, meanwhile, are seen as the worst of the platforms when it comes to acting on content.
24: EU leaders agree on first ever economic sanctions against Belarus.
24: The European Parliament approves the first ever EU climate law. Green and left‐wing MEPs vote against the bill as not being ambitious enough.
24: MEPs pass a resolution demanding the right to legal and safe abortions. The non‐binding legislation notes that women had too many constraints when it came to sexual and reproductive health and rights. The vote came after British‐overseas territory Gibraltar decided to ease its tough anti‐abortion laws, even as Poland recently tightened its harsh regime.
28: Swedish prime minister Stefan Löfven resigns after vote of no confidence. Sweden's speaker of parliament tasks Ulf Kristersson, the leader of the right‐wing opposition's largest party, to form new government.
28: Belarus recalls its ambassador from Brussels in reaction to EU economic sanctions.
29: A group of MEPs call for the suspension of EU funds to Slovenia following Ljubljana's failure to appoint candidates to the EPPO.
29: MEPs and the European Council agree on the new EU Asylum Agency (EUAA), which will replace the existing European Asylum Support Office.

**July**

1: Turkey withdraws from the Istanbul Convention. This is the first time a Council of Europe member has withdrawn from an international human rights convention.
1: The Commission adopts Slovenia's recovery plan (€1.8 billion in grants, €705 million in loans), as the country commences it Presidency of the EU Council. Slovenia formally assumed the six‐month rotating position amid criticism of its conservative PM Janez Janša, who is seen as following Hungary and Poland in undermining the rule of law and democratic values in the EU.
1: The EU‐wide Covid‐19 certificate, a document consisting of a QR code, comes into force.
2: A group of MEPs from the liberal Renew Europe group urge the Commission in a letter to reject Hungary's Covid‐19 recovery plan over concerns about fraud, corruption and LGBTQI rights.
5: A new Eurobarometer survey reveals that for the first time European citizens consider climate change as the single most serious problem facing the world, even more than the Covid‐19 pandemic.
5: EU Council president Charles Michel pledges solidarity to Lithuania after 560 migrants crossed the border from Belarus during the previous weekend.
6: French NGO Reporters Without Borders names Hungarian PM Viktor Orbán, the only EU leader, in a new report, among 37 heads of state who are ‘press‐freedom predators’.
7: Swedish PM Stefan Löfven is voted back into office, one week after he resigned.
8: Spanish NGO Caminando Fronteras reports some 2,087 people died between January and June this year trying to reach Spain from Africa by sea.
8: Belarus expels the head of mission at the Lithuanian embassy in Minsk and the consul general in the town of Grodno, prompting Lithuania to take similar action.
8: Commission president Ursula von der Leyen voices EU's rejection of Turkey's two‐state solution on the Cyprus conflict.
9: The Tokyo Olympics organizers announce the games will take place without spectators throughout their duration, as a resurgent coronavirus forced Japan to declare a state of emergency in the capital.
11: The PAS party of pro‐Western Moldovan president Maya Sandu wins early parliamentary elections.
11: Elections in Bulgaria end with another inconclusive result, with GERB party of former PM Boyko Borissov marginally losing against the new anti‐establishment “There Is Such a People” (ITN) party of Slavi Trifonov.
13: Thousands of French citizens rush to book a Covid‐19 vaccination appointment, after president Emmanuel Macron warned that unvaccinated people would be refused access to a variety of events and venues. He also added that vaccines would become mandatory for health and care workers, with career‐ending penalties for those who refuse to get the jab by mid‐September.
13: EU finance ministers approved investment plans from 12 member states, paving the way for the first payments of grants and loans from the €800 billion Covid‐19 recovery fund to boost the economy.
14: France's anti‐trust watchdog fines Google €500 million for using material from French news agencies.
14: Crowds attending France's annual Bastille Day parade were required to show Covid vaccine papers, documents of a recent infection, or negative test results and wear masks. A few hundred anti‐mask protesters chanted ‘Liberté!’ and scuffled with police, who fired teargas.
20: The EU, the UK and NATO accuse China of carrying out a cyberattack on Microsoft exchange email servers earlier this year that affected almost 30,000 organizations globally.
20: The Commission proposes a new set of anti‐money laundering and counter terrorism financing rules. The legislative package includes the creation of a new EU‐level Anti‐Money Laundering Authority, which will seek to co‐ordinate with national authorities to ensure the rules are applied consistently throughout the private sector.
20: The UK will pay France €62.7 million to prevent migrants from crossing the English Channel, in a deal that includes doubling the number of French officers deployed along the coast.
22: In response to criticism from the EU, Hungarian PM Viktor Orbán announces a referendum on his country's controversial new anti‐LGBTQI law.
24: The US and Germany agree on Nord Stream 2 deal to prevent Russian gas from being used as political leverage by Moscow over Europe. The deal sees Ukraine receiving €42.3 million in green energy technology credits and a guarantee of repayment for gas transit fees it will lose by being bypassed by the pipeline through 2024.
26: Hundreds of Hungarians protest in Budapest against the alleged surveillance carried out by their government with the Israeli‐made Pegasus spyware.
27: Lithuania reports the detention of 171 migrants trying to enter from Belarus, the highest number in a single day so far this year.

**August**

2: NGO ships rescue approximately 800 people in the central Mediterranean over the past few days. A spike in pushbacks by the Libyan coastguard is detected, as more boats are found in distress.
2: Twitter announces it is collaborating with Reuters and the Associated Press (AP) to tackle disinformation.
2: The EU imposes sanctions on Nicaragua's first lady (who is also vice‐president) Rosario Murillo, plus seven other individuals responsible for serious human rights violations.
2: Vitaly Shishov, who led NGO helping Belarusian exiles (Belarusian House in Ukraine), is found dead in Kyiv one day after being reported missing.
4: Belarusian athlete Krystsina Tsimanouskaya flies to Poland after she was granted a humanitarian visa earlier this week. The International Olympic Committee has launched an official investigation into allegations over how her team tried to force her to return home from Tokyo against her will.
4: The Commission announces a preliminary deal for the purchase of 200 million Covid‐19 vaccine doses by the US pharmaceutical company Novavax.
9: Italy's former prime minister Giuseppe Conte is officially elected leader of the 5‐Star Movement.
9: France and Italy face mass protests against the Covid pass, which protestors maintain infringe on their civil liberties.
10: The Polish Border Guard reports a record number of migrants crossing the Polish border from Belarus. Latvia declares a state of emergency along its border and Lithuania decides to erect a fence in new measures to deter migrants they say Belarus is encouraging to cross illegally.
14: Thousands join LGBTI pride march in Bucharest ahead of planned legislation targeting minority rights.
15: European countries scramble to evacuate their citizens and Afghan staff from Kabul. The US troops kept the Afghan capital's airport open as the country was suddenly taken over by Taliban extremists.
16: The death toll in northern Turkey rises to 70 in the wake of this month's devastating floods.
18: Russia's 2021 wildfires are already its largest in the history of satellite observations, Greenpeace Russia reports.
18: Spain and Lithuania receive their first tranche of money from the €800 billion EU recovery fund.
18: Some 12 Belarusian officers armed with riot‐control gear cross the border into Lithuania to force 35 irregular migrants into the country.
20: The Greek government seeks help from abroad to replace its ground firefighting teams which are at the brink of exhaustion after three weeks of battling massive wildfires. More than 500 wildfires have broken out in recent weeks across Greece as the country grapples with its worst heatwave in over 30 years.
20: Greece, Cyprus and Israel affirm Mediterranean alliance amid Turkey's oil exploration exploits in the Eastern Mediterranean.
24: Poland becomes the latest European country to start building a new anti‐refugee fence on its border with Belarus.
25: *The Guardian* reports British tourists facing difficulties in proving their vaccine status in Europe following a delay in linking the British National Health Service's (NHS) Covid pass to the EU's system.
25: The EU and the US sound the alarm over the recent deployment of troops from Eritrea to the Ethiopian region of Tigray, after nine months of war, with thousands of casualties and an increasingly serious humanitarian crisis.
27: Workers at public hospitals in Greece hold a five‐hour work stoppage to protest a government decision making vaccination against Covid‐19 mandatory for all healthcare workers in the public and private sector.
29: The Italian coastguard collects 539 people from a fishing boat drifting near the small Mediterranean island of Lampedusa in one of the largest single rescue operations on record.

**September**

1: The Commission reports that almost 70 per cent of adults in the EU are now fully vaccinated against Covid.
1: Poland declares a state of emergency in a 3 km‐zone along its border with Belarus, giving security forces more powers to keep out migrants being pushed across by Belarusian authorities.
1: Support for EU accession has dropped in Western Balkan countries after member states vetoed opening talks with North Macedonia, Serbian president Aleksandar Vučić says at a forum in Slovenia.
1: The ECDC states that people who already had two anti‐Covid jabs do not need further booster shots, although specific categories of individuals could benefit from it.
2: The WHO begins monitoring a new coronavirus variant called ‘Mu’.
2: Ireland data privacy watchdog fines Facebook‐owned messaging app WhatsApp €225 million for infringements of EU data protection rules.
3: The EU and AstraZeneca reach a settlement agreement on vaccine supplies, ending the pending litigation.
3: EU's foreign policy chief, Josep Borrell, states that the EU wants to engage with, but not recognize, the Taliban as part of a wider effort to evacuate more Europeans and Afghan nationals.
7: Germany accuses Russia of another cyberattack on its parliament.
7: The EU prepares a fifth round of sanctions against Belarus over its recent campaign of pushing migrants across EU borders.
8: A trial of unprecedented scale starts for 20 men suspected of involvement in jihadist attacks across Paris in November 2015, the deadliest attack in France's peacetime history.
10: ECtHR rejects a request from Greek healthcare workers to apply a pause to their mandatory Covid‐19 vaccination.
10: Russia and Belarus begin the largest military drill seen in Europe in decades, with the ‘Zapad 2021’ exercise – more than 200,000 troops will be mobilized in a simulated war with NATO powers.
14: A court in Amsterdam rules in favour of Uber drivers in the Netherlands being entitled to the same employment benefits as taxi drivers.
14: Turkey refuses to release jailed human rights defender Osman Kavala despite a ruling by the ECtHR.
15: France receives Commission approval to pay €3 billion in state aid (Transition Fund) to approximately 100 companies in distress due to the pandemic.
15: Romanian socialist MEPs join right‐wing nationalist Polish and Hungarian counterparts in voting against LGBTQI rights.
15: Commission President Ursula von der Leyen launches Europe's ‘Global Gateway’ strategy, an international investment plan for transport and infrastructure which comes as a clear response to China's Belt and Road Initiative (BRI).
16: Eurozone wages fall for the first time in a decade, dropping 0.1 per cent since June 2020.
16: Bulgarian president Rumen Radev's re‐appointed incumbent caretaker premier Stefan Yanev is appointed lead in interim administration until a new government is formed.
17: The European Parliament supports the creation of a new EU ethics body which will help tackle and prevent conflicts of interest, revolving doors and corruption inside EU institutions.
17: The Commission blocks the recently‐created EPPO from using their budget to hire necessary specialised personnel – no explanation was provided.
17: EU Commission Vice‐President Margaritis Schinas announces the launch of a new health agency which will help prepare for future pandemics. The ‘Health Emergency Preparedness and Response Authority’ (HERA) will assess threats, fund research and build stockpiles of drugs.
19: Poland confirms the first fatalities among people trying to enter the EU via Belarus.
20: The Netherlands opposes any new EU legal framework on voluntary returns for rejected asylum seekers, in fear of this giving them more rights.
21: The ECJ orders Poland to pay a daily fine of €500,000 for not ceasing lignite extraction activities at Turow mine.
22: The EU Agency for Fundamental Rights reports that the pandemic has exacerbated the day‐to‐day challenges that civil society faces, whereby many organizations saw their work conditions deteriorate.
22: The Polish region of Swietokrzyskie votes to abandon an anti‐LGBTQI declaration from 2019, after the EU threatened to withhold pandemic recovery funding.
23: EU states, 37 MEPs and the Commission sign a transparency pledge as part of the Conference on the Future of Europe, to ensure that the EU's decision‐making process becomes more open to a wider public.
23: Italian police arrest Catalan independence leader and MEP Carles Puigdemont at an airport in Sardinia. He is to appear in court the next day and could be extradited to Spain, on charges of ‘sedition’.
24: Ukraine's parliament passes a law ordering oligarchs to register and stay out of politics.
26: Approximately 64 per cent of Swiss people vote to legalize same‐sex marriage in a referendum.
26: Abortion becomes legal in San Marino, after 73 per cent of its people voted for this in a referendum.
27: Dutch PM Mark Rutte fires his economy minister, Mona Keijzer, after she criticized his vaccine‐pass policy in a newspaper interview.
28: The European Parliament formally nominates Russian dissident Alexei Navalny for this year's ‘Sakharov’ human rights prize.
30: The EU is expected to send over 100 election monitors to a regional vote in Venezuela after being invited to do so by the country's National Electoral Council. This comes as a sign of potential detente between the Venezuelan regime and Europe.

**October**

1: Amnesty International satellite images and other data indicate that Polish officers appear to have forced a group of 32 Afghan asylum seekers across the border from Poland to Belarus in what was an ‘unlawful pushback’. A senior Polish government official conceded that migrant children, including infants, were among those authorities had driven back to the Belarusian border.
4: The weekly trend of Romania's fourth wave of Covid‐19 put the country ahead of all other EU member states and sixth worldwide.
4: Czech billionaire PM Andrej Babiš and Cypriot president Nicos Anastasiades face inquiry after their shady dealings came to light in the latest financial data leak (‘Pandora Papers’).
4: The ECDC warns countries with low vaccination rates of ‘a significant surge in [Covid] cases, hospitalisations, and mortality for the upcoming two months … due to very high virus circulation’.
4: BepiColombo, a probe launched by the European Space Agency, sends back its first photographs of Mercury.
5: An Italian judge in Sardinia delays Spain's extradition request on Puigdemont.
5: Facebook blames a ‘faulty configuration change’ for a nearly six‐hour outage that prevented the company's 3.5 billion users from accessing its social media and messaging services.
5: The Commission unveils its first ever strategy to tackle antisemitism and promote Holocaust remembrance. Under the proposal, the EU executive aims to create a Europe‐wide network of experts and industry representatives who can remove illegal online hate speech. The EU will also develop a European research hub on contemporary antisemitism and Jewish culture.
5: Romania's liberal government is ousted following a no‐confidence vote overwhelmingly endorsed by parliament.
6: After a report found that at least 330,000 children were victims of sexual abuse by clergy and lay members over 70 years, the French Catholic Church has asked for forgiveness after admitting the findings of ‘appalling’ abuse.
7: Hungary and Poland veto EU strategy paper at the EU Council on children's rights.
11: Austrian chancellor Sebastian Kurz resigns over allegations his ÖVP party misused state funds to manipulate opinion polls in its favour – Alexander Schallenberg is sworn in as his replacement.
12: Russia's reduced gas flows via Ukraine to Moldova prompt Moldova's new, pro‐EU government to seek EU help.
12: The EU launches its first ever green bond. The 15‐year green bond, which will raise €12 billion, received more than €135 billion in demand, making it the largest green‐bond launch and the highest level of demand for a green bond sale to date.
13: Frontex head, Fabrice Leggeri, reveals reports detailing an increase in alleged violations against migrants in Lithuania.
13: Commission unveils a set of proposals aimed at easing bureaucracy and checks on post‐Brexit trade in Northern Ireland.
14: Belarus threatens to jail readers of banned media with up to seven years under a new decree signed earlier in October.
14: Slovenian PM Janez Janša, the current EU presidency‐holder, tweets a picture of 13 MEPs whom he accused of being ‘puppets’ of Hungarian‐born, American‐Jewish philanthropist George Soros.
15: A court in Naples sentences the captain of the Asso28 tugboat to a year in prison for disembarking over 101 rescued migrants in Libya's capital city Tripoli – this is the first such sanction in Italy and in Europe.
15: Polish MPs vote to let officials immediately expel asylum seekers who entered Poland irregularly (which is forbidden under international law) and to build a €350 million wall on the Belarusian border.
18: EU sends emergency Covid aid to Romania.
18: Roberto Gualtieri, for the centre‐left Democratic Party, wins 60 per cent of the vote in Rome's mayoral election. He will succeed Virginia Raggi, a politician with the populist 5‐Star Movement (M5S).
19: Conservative‐liberal mayor Péter Márki‐Zay wins the first ever primary elections in Hungary. Six opposition parties organized this vote in order to have one united opposition figure run against nationalist PM Orbán and his Fidesz candidates.
29: The UK unveils its national strategy to reach net‐zero emissions by 2050, ahead of COP26 climate talks in Scotland.
20: Poland doubles its troops to 6,000 on its Belarus border.
20: The sharp increase in Covid‐19 infections in eastern EU member countries, particularly the Baltic states, puts health systems under increasing pressure, prompting governments to reimpose restrictions.
21: Kremlin critic Alexei Navalny wins the Sakharov Prize for defending human rights.
22: Britain charges with terrorism the man alleged to have stabbed to death MP David Amess last week. British prosecutor James Cable states that the accused ‘considered himself affiliated to Islamic State’.
24: German police stop some 50 armed far‐right vigilantes on the Polish‐German border. The group had gone there to keep out migrants coming in via Belarus and Poland.
25: Hungarian PM Viktor Orbán accuses the EU and US of trying to help unseat him in next year's elections.
26: The Commission demands the immediate release of the detained Sudanese leadership, including its PM and cabinet members.
27: The ECJ orders Poland to pay a €1 million‐per‐day fine for not suspending the disciplinary chamber of its Supreme Court, which has been ruled a violation of EU law.
27: The Italian senate votes by 154 to 131 to block a new law criminalizing homophobic and misogynist attacks as well as violence against disabled people. Far‐right parties had previously campaigned against this bill and the Vatican had also lobbied in the same direction.
28: The Commission agreed a €60 million grant for Moldova to help its pro‐EU government deal with an energy crisis linked to shrewd Russian bargaining.
29: The European Parliament launches a lawsuit against the Commission at the CJEU for failing to use a ‘rule‐of‐law conditionality mechanism’ on withholding EU funds for countries such as Hungary and Poland.
31: Pro‐EU North Macedonia's PM Zoran Zaev steps down after his Social Democrat party lost in local elections including in Skopje.
31: A cargo ship with some 382 Afghan refugees arrives in Greece following a four‐day standoff with Turkey. This is the largest single number of arrivals in the country in years.
31: The US and EU agree to end a trade dispute on steel and aluminium tariffs at the G20 summit in Rome.

**November**

2: France and the UK issue rival ultimatums in a dispute on fishing rights as talks continue to avoid a trade war.
2: The Commission adopts new cybersecurity rules for wireless devices with the aim of preventing online payment fraud and better protecting citizens' personal data.
3: EU states' soldiers commence a two‐year long mission to train 11 special forces units from Mozambique's military and help the country repel Islamist insurgents.
4: Czech centrist and centre‐right parties reach an agreement on forming a majority coalition government and its key agenda.
5: Romania records its highest daily number of Covid‐19 deaths since the pandemic started (almost 600 dead in only 24 hours).
5: MPs from the Nordic states agree mutual defence on cyberattacks at their summit.
5: More than 1,000 universities and science academies in Europe urge the Commission to let Britain expeditiously join its €95.5 billion ‘Horizon’ research programme, following 10 months of Brexit‐linked delays.
5: Reuters reports that Lithuania, for the past week, started erecting a 3.4 metre high steel fence topped with 0.6 metres of razor wire along its shared land border with Belarus.
6: Thousands of protesters march against a recent anti‐abortion law in several Polish cities following the death of a woman, Izabella, after doctors declined to intervene in her pregnancy complications.
8: Germany's Covid‐19 infection rate rises to 201 per 100,000, its highest level since the start of the pandemic.
8: France's bishops agree to selling part of the Church's extensive real estate holdings, in order to compensate victims of decades of child sexual abuse by the clergy.
8: Belgium urges people to start working from home again due to surging Covid infections.
10: The Missing Migrants Project run by the International Organization for Migration (IOM) reports that more people crossing the Mediterranean Sea to reach Europe have died so far this year (2,457 deaths and/or missing persons) than any year since 2018 (2,344).
10: Google loses a crucial appeal in an anti‐trust case launched by the European Commission at the EU General Court. The company received a €2.42 billion anti‐competition fine.
10: Czech prosecutors ask the newly elected lower house of parliament to lift PM Andrej Babiš's immunity from prosecution over alleged fraud involving EU funds.
12: Austria, Estonia, Latvia, Lithuania and Poland appeal to the Commission for migrant relief on the EU‐Belarussian border.
14: A new centrist, anti‐corruption party called ‘We Continue The Change’ (PP) wins Bulgarian elections with 26 per cent of the vote, ahead of GERB.
15: Austria instructs its approximately 2 million unvaccinated citizens to stay home, except for essential work or food‐shopping trips – this is the EU's first ever selective lockdown of its type.
17: Greece offers to loan UK museums two classical artefacts, the golden Mask of Agamemnon and the Artemision Bronze statue of Zeus should Britain permanently return the Elgin Marbles to Athens.
17: Hungary reports 10,265 new Covid‐19 infections, its highest daily tally since the end of March.
17: Belarus begins moving some 1,000 migrants from a camp on the Polish border to a temporary shelter.
19: The Polish army detains hundreds of migrants who crossed the Belarus border and accused Belarusian special forces of masterminding the operation. Around 2,000 people, mainly from the Middle East, are estimated to be living rough near the Polish‐Belarus border in dire conditions.
19: ‘Doctors without Borders’ (MSF) rescue boat GeoBarents seeks to disembark 186 people it has rescued in the Mediterranean Sea. A boat carrying the bodies of 10 migrants, who were believed to have suffocated in overcrowded conditions, arrives in Sicily.
19: Bulgarian president Rumen Radev wins a second term in office by a wide margin.
19: The Slovenian government finally decides to nominate two delegated prosecutors for the EPPO after a long delay.
23: Three candidates from the centre‐right European People's Party (EPP), Austrian MEP Othmar Karras, Maltese MEP Roberta Metsola and Dutch MEP Esther de Lange, launch their bids for the next European Parliament presidency.
23: MEPs vote in favour of the new Common Agriculture Policy (CAP) for 2023–27, without the support of Green lawmakers.
24: The ECDC recommends that EU countries should consider booster doses for all adults, with priority for those above 40 years old, to limit the spread of the virus and reduce the risk of a ‘very high burden’ on healthcare systems.
25: The AFP reports more than 30 people are feared dead after a migrant boat capsized in the English Channel, the deadliest disaster on the route to date.
26: A surprise new cabinet bringing together the former political foes of the National Liberal Party (PNL) and the Social Democrat Party (PSD) is sworn in by the Romanian president, Klaus Iohannis.
26: Commission president Ursula von der Leyen tweets that the EU will block travel from South Africa due to a new variant of Covid‐19 there.
28: France, Belgium, the Netherlands and the EU Commission agree in talks in Calais for Frontex to fly a surveillance plane ‘day and night’ over the English Channel and help spot migrant boats.
28: Swiss people vote to keep coronavirus vaccine passes and other restrictions in a referendum.
28: Czech president Miloš Zeman appoints the leader of a centre‐right alliance Petr Fiala as PM.
29: Swedish Social Democratic party leader Magdalena Andersson, who in the previous week became Sweden's first female PM for seven hours before resigning after a budget defeat saw a coalition partner quit, is elected again as the country's head of government.
29: Greece opens two new EU‐funded ‘closed controlled structures’ for migrants, with barbed wire fences and CCTV, on the islands of Kos and Leros.
30: Poland's president signs legislation that will limit the access of aid charities and journalists to its border with Belarus.

**December**

1: Frankfurt's higher regional court finds a 29‐year‐old guilty of genocide and crimes against humanity resulting in death, sentencing him to life in prison. The case is the first in the world to decide whether a former member of the so‐called Islamic State group played a role in the attempted genocide of the Yazidi religious group.
1: The International Energy Agency (IEA) reports ‘record year of growth’ for renewable energy, despite the pandemic and rising costs for raw materials.
1: The Commission reveals a proposal to digitalize EU cross‐border justice systems, aiming at making them more accessible and effective. The new draft law aims to tackle inefficiencies affecting cross‐border judicial co‐operation and barriers to access to justice in cross‐border cases.
2: The German government decides to bar non‐vaccinated people from entering businesses other than essential grocery stores and pharmacies.
2: The ECDC warns that the Omicron variant is expected to be detected in over half of all cases of Covid‐19 in Europe within the next few months.
6: Hungary and Estonian diplomats block a revised set of EU tax rules, potentially derailing an effort to curb the race for ever‐lower corporate taxes in the EU.
6: The European Defence Agency (EDA) submits in a report that EU countries spent their highest level ever recorded on defence in 2020.
6: The chairman of Slovenia's anti‐corruption commission, Robert Šumi, warns that the country loses €3.5 billion a year due to corruption.
6: EU labour ministers agree on minimum standards to better protect minimum wages in Europe. Although the EU draft law will not set a common EU‐wide minimum wage, this new legislation is seen as a potential game‐changer in countries where salaries are very low.
7: EU finance ministers agree on a new set of rules for value‐added tax (VAT) consumers and businesses pay for products and services.
7: Finance ministers from Hungary and Estonia reject updated Code of Conduct for tax rules.
7: Suspected hitman in Khashoggi murder is arrested in Paris.
9: The Commission is recommending police be allowed to arrest and, if necessary, shoot suspects in other member states.
9: The Bundestag elects Olaf Scholz as the new German chancellor, in succession to Angela Merkel.
9: The Commission unveils its legislative proposal for improving the working conditions of so‐called platform or gig workers, like Uber drivers or Deliveroo riders.
9: The Commission lays out plans for a legal basis to criminalize hate speech and hate crime at EU level, paving the way for Europe‐wide sanctions on hate‐motivated crimes. The initiative is particularly aimed at protecting women and the LGBTIQ.
9: The European Consumer Organisation (BEUC) welcomes an agreement on extending and updating roaming rules.
10: EU governments agree that Croatia is ready to join the passport‐free ‘Schengen’ travel zone.
15: The Commission presents a proposal, tightening environmental criminal law, that clarifies current definitions of environmental crime offences, adding new provisions for the illegal timber trade, illegal ship recycling or illegal abstraction of water.
16: The incoming Dutch cabinet, to be led for a fourth time by PM Mark Rutte, reaches a coalition agreement.
16: EU leaders agree to impose further economic sanctions on Russia if it invaded Ukraine.
17: According to the Danish Refugee Council (DRC) almost 12,000 prospective asylum seekers in Europe were illegally forced back across a border in 2021.
18: Dutch prime minister Mark Rutte announces a sudden hard lockdown.
20: The Commission approves the use of the vaccine developed by Novavax after a positive scientific recommendation from the EMA.
21: Amnesty International documents and reports numerous beatings by Belarus guards of migrants after being forced back into Belarus from EU states.
21: The annual Davos World Economic Forum (WEF) planned for January is postponed amidst increasing concerns over the coronavirus Omicron variant.
21: PM Victor Orbán states that Hungary will not change its controversial migration laws despite a ruling by the ECJ.
22: UN migration officials report that more than 160 people drowned in two separate shipwrecks off the coast of Libya in the past week.
22: Denmark is to rent 300 prison cells from Kosovo to house non‐EU prisoners convicted in Denmark and awaiting deportation to their home countries after serving their sentences.
22: In an escalation of the rule‐of‐law row, the Commission launches an infringement procedure against Poland over the recent ruling by the country's controversial Constitutional Tribunal, which put into doubt the primacy of EU law.
22: Naftogaz, the largest natural gas company in Ukraine, files a complaint with the EU competition watchdog against Russia's Gazprom. The Ukrainian company accuses Gazprom of abusing its dominant position in the European gas market.
23: Bulgarian PM Kiril Petkov announces that Bulgarian pensioners who get their first or second vaccine dose in the next six months will get a one‐off payment of about €40.
24: Almost 800 people on three NGO rescue vessels remain stranded at sea in the Mediterranean ahead of the Christmas holidays.
24: An Italian court drops investigations related to charges of aiding illegal migration against Carola Rackete, the former captain of German rescue vessel Sea‐Watch 3.
25–26: German NGO Sea‐Watch rescues 446 migrants from the Mediterranean Sea in five operations over the holiday weekend.
27: Belarus authorities release a draft document proposing amendments to the country's constitution, which may allow President Alexander Lukashenko to remain in office until 2035.
29: France announces that its Covid infection cases soared to 208,000, a record number in Europe for the second day in a row. Starting 31 December, wearing masks outdoors will become mandatory in Paris.
31: Data from the Friedrich Loeffler Institute (FLI) show Europe is facing the worst bird‐flu outbreak ever.
31: According to Reuters, coronavirus deaths in eastern Europe exceed 1 million.

